# P04.43. Children with common neurological conditions use complementary and alternative medicine twice as frequently as those without

**DOI:** 10.1186/1472-6882-12-S1-P313

**Published:** 2012-06-12

**Authors:** L Jansons, K James, J Ziegenfuss, J Tilburt

**Affiliations:** 1 Mayo Medical School, Rochester, USA

## Purpose

Recent literature suggests that one in nine children in the US uses some type of complementary and alternative medicine (CAM). Children with challenging neurological conditions such as headaches, migraines, and seizures may seek CAM in their attempts at self-care. Our objective was to describe CAM use in children with these conditions.

## Methods

We compared use of CAM in 9,417 children with and without common neurological conditions (headaches, migraines, seizures), using the 2007 National Health Interview Survey (NHIS) data, where CAM might plausibly play a role in their self management.

## Results

Children with common neurological conditions reported significantly more CAM use compared to the children without these symptoms (22.2% vs 11.0%, p<0.0001). Compared to other pediatric CAM users, children with neurological conditions report significantly higher use of mind-body techniques than those without (36.6% vs 20.5%, p<0.007). Of the mind-body techniques, deep breathing (33.7%), meditation (13.3%), and progressive relaxation (9.4%) were used most frequently.

## Conclusion

CAM use is twice as common in children with common neurological conditions compared to those without. More work is needed to further characterize the nature of CAM use in this population as well as its benefits on neurological disease. Whether and how to harness the self-management benefits of some CAM modalities in the care of pediatric patients with neurological conditions is an important area of investigation.

